# Efficacy of Sodium‐Glucose Cotransporter‐2 Inhibitors in Patients With Acute Myocardial Infarction: A Meta‐Analysis of Randomised Controlled Trials

**DOI:** 10.1002/edm2.514

**Published:** 2024-08-15

**Authors:** Mushood Ahmed, Hritvik Jain, Hira Javaid, Areeba Ahsan, Szabolcs Szilagyi, Adeel Ahmad, Raheel Ahmed

**Affiliations:** ^1^ Rawalpindi Medical University Rawalpindi Pakistan; ^2^ All India Institute of Medical Sciences (AIIMS) Jodhpur India; ^3^ Allama Iqbal Medical College Lahore Pakistan; ^4^ Foundation University Medical College Islamabad Pakistan; ^5^ Department of Cardiology Northumbria Hospitals NHS Foundation Trust Newcastle Upon Tyne UK; ^6^ Chelsea and Westminster Hospital London UK; ^7^ Department of Cardiology Royal Brompton Hospital London UK; ^8^ National Heart & Lung Institute Imperial College London London UK

**Keywords:** dapagliflozin, empagliflozin, myocardial infarction, sodium‐glucose co‐transporter inhibitors

## Abstract

**Background:**

Sodium‐glucose cotransporter 2 (SGLT2) inhibitors improve cardiovascular (CV) outcomes in patients with or without Type 2 diabetes and heart failure (HF). However, studies have shown conflicting evidence regarding their efficacy in patients following acute myocardial infarction (MI). We conducted a pilot systematic review and meta‐analysis to synthesise the available evidence regarding the effectiveness of SGLT2 inhibitors in MI.

**Methods:**

A systematic literature search was conducted using PubMed/MEDLINE, the Cochrane Library and Embase databases to identify randomised controlled trials (RCTs) that compared clinical outcomes of SGLT2 inhibitors with placebo following MI. We conducted the statistical analysis using RevMan, version 5.4 and pooled risk ratios (RRs) along the corresponding 95% confidence interval (CI) for all outcomes.

**Results:**

Five RCTs reporting data for 11,211 patients were included in our study. The mean follow‐up duration was 43.8 weeks. Our pooled analysis showed that SGLT2 inhibitors significantly reduced the risk of hospitalisations for heart failure (HHF) (RR = 0.76, 95% CI: 0.61–0.88, *p* = 0.001) in patients with MI. However, the risk of all‐cause mortality (RR = 1.05, 95% CI: 0.78–1.41, *p* = 0.76), CV mortality (RR = 1.04, 95% CI = 0.84–1.29, *p* = 0.73) and all‐cause hospitalisations (RR = 1.06, 95% CI: 0.96–1.17, *p* = 0.25) remained comparable across the two groups.

**Conclusion:**

SGLT2 inhibitors reduce HHF without affecting all‐cause mortality, CV mortality and all‐cause hospitalisations. However, further evidence is required to reach a definitive conclusion.

## Graphical abstract

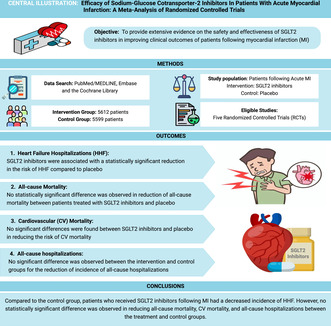

**Summary**

- This meta‐analysis of 11,211 patients demonstrates the efficacy of SGLT2 inhibitors in reducing hospitalisations for heart failure in patients presenting with acute myocardial infarction.
- The risk of all‐cause mortality, cardiovascular mortality and all‐cause hospitalisations remained comparable across the treatment and control groups.
- Large‐scale, RCTs with better representation of females, along with longer follow‐ups are warranted to evaluate the robustness of SGLT2 inhibitors in this population.

## 1 Introduction

Patients with myocardial infarction (MI) are at an increased risk of heart failure (HF), especially if associated with congestion or reduced left ventricular ejection fraction [1, 2]. Sodium‐glucose cotransporter 2 (SGLT 2) inhibitors, particularly dapagliflozin and empagliflozin, have demonstrated efficacy in improving clinical outcomes like cardiovascular (CV) mortality, and hospitalisations for heart failure (HHF) in patients with or without Type 2 diabetes and HF [3]. Studies have shown that the use of SGLT 2 inhibitors following MI led to improved myocardial function and cardiac sympathetic activity [4, 5, 6]. However, the EMPACT‐MI trial (Study to Evaluate the Effect of Empagliflozin on Hospitalization for Heart Failure and Mortality in Patients with Acute Myocardial Infarction) which enrolled 6522 MI patients showed non‐significant improvement in CV outcomes on treatment with empagliflozin [7]. Considering these conflicting findings, we conducted a pilot systematic review and meta‐analysis to investigate the efficacy of SGLT2 inhibitors in patients following acute MI, by comprehensively analysing data from published randomised controlled trials (RCTs).

## 2 Methods

The systematic review and meta‐analysis followed the guidelines established by the Preferred Reporting Items for Systematic Review and Meta‐Analysis (PRISMA) [8]. We registered the protocol of review with PROSPERO (CRD42024537970).

We systemically searched PubMed/MEDLINE, the Cochrane Library and Embase databases using broad search keywords (“SGLT2 inhibitors”, “empagliflozin”, “dapagliflozin”, “canagliflozin”, “Acute myocardial infarction” and “MI”) to identify eligible RCTs that evaluated the safety and efficacy of SGLT2 inhibitors following MI.

The studies were included if they were published RCTs with a follow‐up duration of at least 12 weeks, included adult participants (age ≥ 18) with acute MI, compared the safety and efficacy of SGLT2 inhibitors with placebo, SGLT2 inhibitors were administered after acute MI and reported at least one of the outcomes of interest. Outcomes included all‐cause mortality, CV mortality, HHF and all‐cause hospitalisations. We excluded observational studies, non‐full text studies, animal studies and reviews.

Two independent reviewers evaluated the articles, and the extracted data encompassed baseline characteristics, inclusion/exclusion criteria and outcomes. We used the Cochrane Risk of Bias tool for randomised trials (RoB 2.0) to assess the quality of each study. The quality of evidence was assessed using the Grading of Recommendations Assessment, Development and Evaluation (GRADE) methodology.

We conducted the statistical analysis using RevMan, version 5.4 and pooled risk ratios (RRs) along the corresponding 95% confidence interval (CI) for all outcomes. Results were pooled using the random‐effects model. We evaluated statistical heterogeneity using the chi‐squared test and the Higgins *I* ^2^ statistic. We also calculated absolute effect sizes per 1000 patients. Significance was determined by a *p*‐value of <0.05 in all cases.

## 3 Results

The systematic literature search yielded 230 records. After screening, five RCTs were eligible to be included in our meta‐analysis. The PRISMA flowchart in Figure S1 depicts the study selection and screening process.

Five RCTs [4, 5, 6, 7, 9] published between 2020 and 2024 were pooled in our study, with 11,211 participants. The treatment group consisted of 5612 patients who received SGLT2 inhibitors, while 5599 patients were in the placebo group. The mean age of the participants was 61.83 years, and the mean follow‐up duration was 43.8 weeks. Three studies used empagliflozin [6, 7, 9], while two used dapagliflozin [4, 5]. The SGTLT2 inhibitor therapy was initiated 3–14 days after acute MI and the follow‐up in the individual studies ranged between 12 weeks and 18 months. The detailed characteristics of the included studies and participants are outlined in Table 1. The inclusion and exclusion criteria of each study are mentioned in Table S1. Four studies had a low risk of bias, while one had some concerns. The Figure S2 provides details of the bias assessment for each included study.

**TABLE 1:** Baseline characteristics of the included studies and participants.

Trial	Country	Sample size SGLT2i/control (*N*)	Timing of study	Dose of SGLT2i	Initiation of treatment	Follow‐up	Age mean ± SD	Males *N* (%)	BMI mean ± SD	Hypertension *N* (%)	Diabetes *N* (%)	Dyslipidaemia *N* (%)	Previous MI‐n (%)
EMBODY 2020	Japan	46/50	February 2018 to March 2019	Empagliflozin 10 mg once‐daily	2 weeks after the onset of AMI	24 weeks	SGLT2i = 63.9 ± 10.4	SGLT2i = 38 (82.6)	SGLT2i = 25.2 ± 3.7	SGLT2i = 38 (82.6)	SGLT2i = 100%	SGLT2i = 34 (73.9)	—
							*C* = 64.6 ± 11.6	*C* = 39 (78.0)	*C* = 25.2 ± 4.1	*C* = 39 (78.0)	*C* = 100%	*C* = 36 (72.0)	—
EMMY 2022	Austria	237/239	11 May 2017 to 3 May 2022	Empagliflozin 10 mg once daily	72 h after PCI	26 weeks	SGLT2i = 57 (52–64)	SGLT2i = 195 (82)	SGLT2i = 27.7 (25.3–30.3)	SGLT2i = 92 (39)	SGLT2i = 30 (13)	SGLT2i = 71 (30)	SGLT2i = 14 (5.9)
							*C* = 57 (52–65)^a^	*C* = 197 (82)	*C* = 27.2 (24.9–30.2)^a^	*C* = 107 (45)	*C* = 33 (14)	*C* = 64 (27)	*C* = 9 (3.8)
DACAMI 2023	Egypt	50/50	From October 2021 to April 2022	Dapagliflozin 10 mg once daily	72 h post‐anterior STEMI	12 weeks	SGLT2i = 55.24 ± 13.2	SGLT2i = 42 (84.0)	SGLT2i = 29.96 ± 4.9	SGLT2i = 32 (64.0%)	SGLT2i = 0	SGLT2i = 7 (14.0)	—
							*C* = 56.70 ± 11.5	*C* = 41 (82.0)	*C* = 30.13 ± 4.6	*C* = 29 (58.0%)	*C* = 0	*C* = 8 (16.0)	—
DAPA MI 2023	Sweden and United Kingdom	2019/1998	December 2020 to March 2023	Dapagliflozin 10 mg once daily	During the hospitalisation for the index MI event or within 10 days from index MI	11.6 months	SGLT2i = 63.0 ± 11.06	SGLT2i = 1631 (80.8)	SGLT2i = 28.2 ± 4.72	SGLT2i = 766 (37.9)	SGLT2i = 0	—	SGLT2i = 178 (8.8)
							*C* = 62.8 ± 10.64	*C* = 1579 (79.0)	*C* = 28.3 ± 4.91	*C* = 716 (35.8)	*C* = 0	—	*C* = 189 (9.5)
EMPACT MI 2024	North America, Latin America, Europe and Asia	3260/3262	December 2020 to March 2023	Empagliflozin 10 mg once daily	Within 14 days after admission	17.9 months	SGLT2i = 63.6 ± 11.0	SGLT2i = females: 812 (24.9)	SGLT2i = 28.1 ± 5.0	SGLT2i = 2262 (69.4)	SGLT2i = 1035 (31.7)	—	—
							*C* = 63.7 ± 10.8	*C* = 813 (24.9)	*C* = 28.1 ± 5.0	*C* = 2276 (69.8)	*C* = 1046 (32.1)	—	—

Abbreviations: BMI, body mass index; *C*, control/placebo; IQR, interquartile range; MI, myocardial infarction; *N*, number; SGLT2i, sodium‐glucose cotransporter 2 inhibitors.

^a^ Data are reported as median and interquartile range.

**FIGURE 1:**
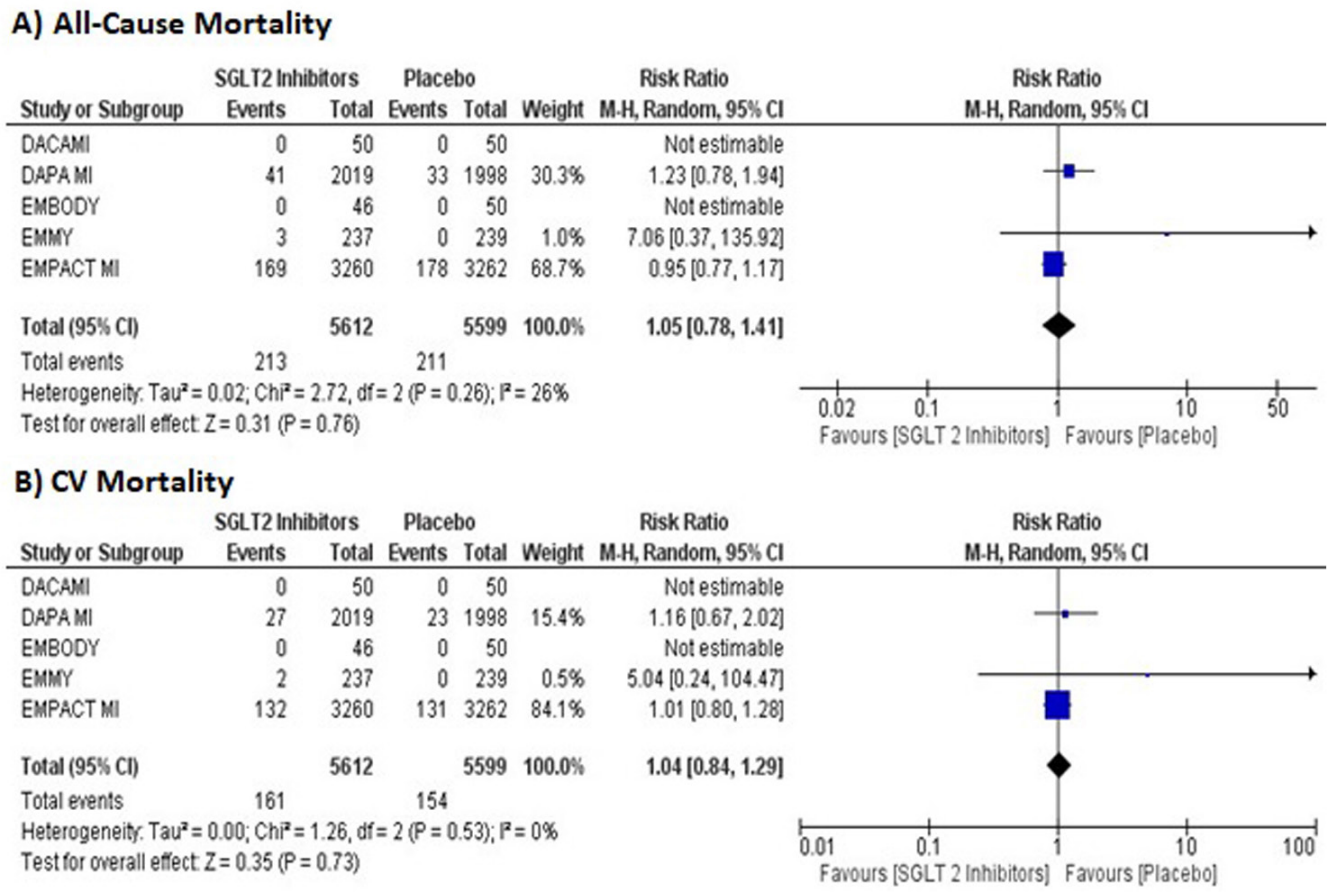
Forest plots for the pooled outcomes: (A) all‐cause mortality and (B) CV mortality. CV, cardiovascular.

The pooled analysis demonstrated no statistically significant difference between SGLT2 inhibitors and placebo in reducing all‐cause mortality (RR = 1.05, 95% CI: 0.78–1.41, *p* = 0.76; high certainty Figure 1A). Low heterogeneity (*I* ^2^ = 26%) was observed across the pooled RCTs.

No significant differences were observed between SGLT2 inhibitors and placebo in reducing the risk of CV mortality (RR = 1.04, 95% CI = 0.84–1.29, *p* = 0.73; high certainty Figure 1B).

**FIGURE 2:**
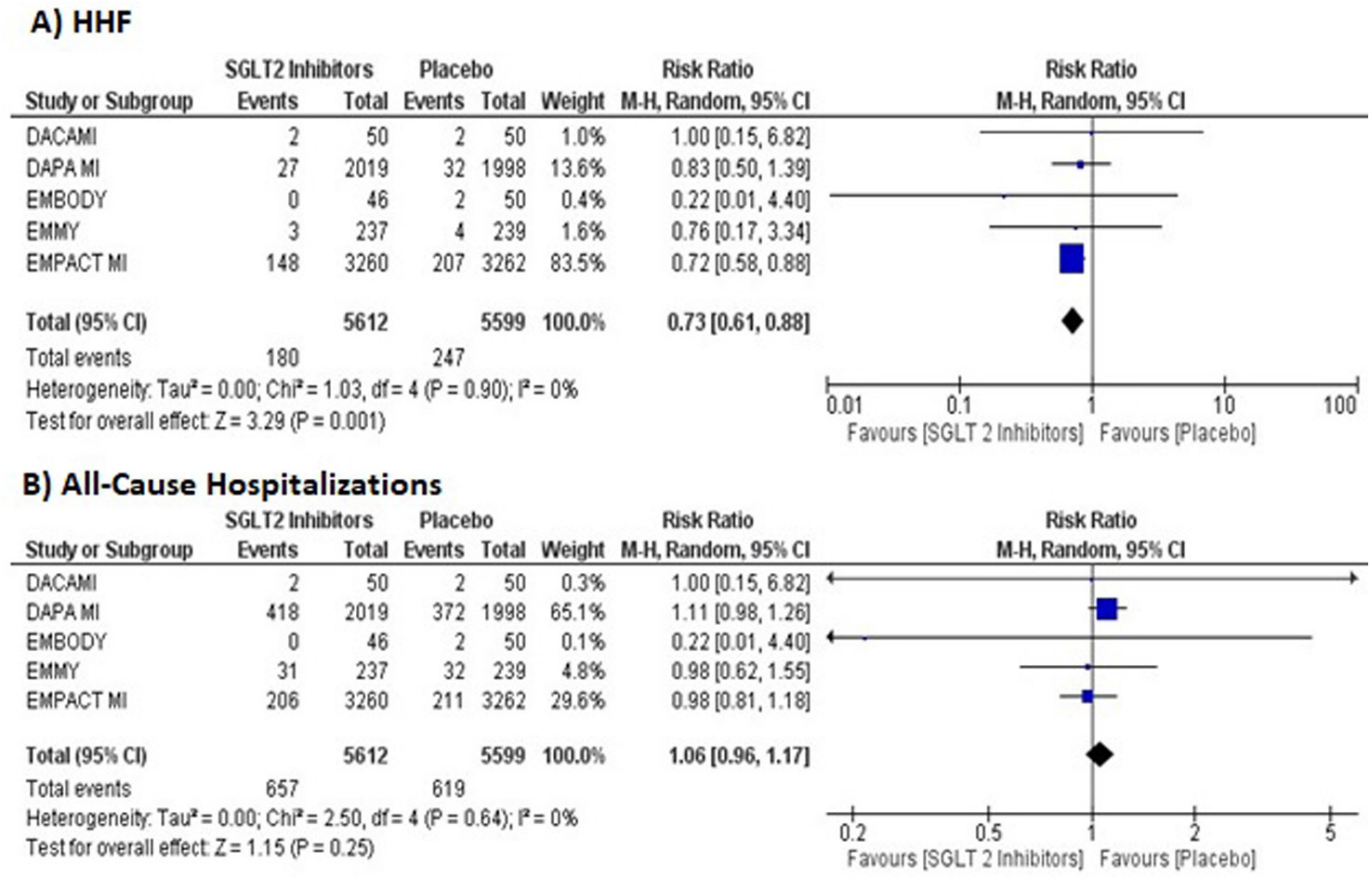
Forest plots for the pooled outcomes: (A) HHF and (B) all‐cause hospitalisations. HHF, hospitalisations for heart failure.

Our pooled analysis showed a significantly reduced risk of HHF in patients treated with SGLT2 inhibitors compared to placebo (RR = 0.76, 95% CI: 0.61–0.88, *p* = 0.001; high certainty Figure 2A). In terms of absolute effects, this translated to 12 fewer HHF per 1000 patients who received SGLT2 inhibitors compared with the placebo (absolute risk difference 12 [95% CI: 17 to 5]; fewer per 1000 patients; Table S2).

The pooled analysis demonstrated no statistically significant difference between SGLT2 inhibitors and placebo for reducing the risk of all‐cause hospitalisations (RR = 1.06, 95% CI: 0.96–1.17, *p* = 0.25; high certainty Figure 2B). No statistical evidence for heterogeneity was found for CV mortality, HHF and all‐cause hospitalisations (*I* ^2^ = 0%).

The meta‐regression could not be performed following the Cochrane guidelines as the pooled studies are <10 and heterogeneity is non‐significant [10]. A similar approach has been adopted by researchers in the past [11].

## 4 Discussion

To the best of our knowledge, this is the first meta‐analysis to date that calculated absolute risk reduction and GRADE assessment of the quality of evidence regarding the efficacy of SGLT2 inhibitors following MI. In this meta‐analysis, high‐quality evidence showed that SGLT2 inhibitors were likely associated with a significantly reduced risk of HHF compared to placebo. However, the risk of all‐cause mortality, CV mortality and all‐cause hospitalisations were comparable between the two groups.

The exact mechanism contributing to a reduction in HHF in MI patients is not yet established. However, the observed reduction in HHF can be attributed to the following factors. SGLT2 inhibitors have been demonstrated to improve both echocardiographic functional and structural parameters [5, 6]. This is associated with the anti‐inflammatory, anti‐oxidative and antifibrotic effects of SGLT2 inhibitors, along with an uninterrupted molecular interaction with the cardiac myocytes that leads to significantly improved myocardial work energetics as well as the simultaneous activation of protective downstream pathways [5, 12]. Empagliflozin also has led to reductions in left ventricular end‐diastolic and end‐systolic volumes in a subset of HF patients without diabetes, which correlates with enhanced left ventricular function. This pathophysiological effect of SGLT2 inhibitors directly affects the myocardium positively [13].

A study by Kwon et al. reported a reduction in all‐cause mortality with the use of SGLT2 inhibitors after MI; however, the relatively shorter duration of follow‐up and the retrospective observational nature of the study are inherently at substantial risk of bias [14]. The EMPACT MI trial [7] included in our pooled analysis did not show improvement in CV outcomes on treatment with SGLT 2 inhibitors; however, there are multiple reasons for these non‐significant findings. The trial was conducted during the COVID‐19 pandemic and HHF decreased substantially during this period. Moreover, two regions that recruited MI patients were affected by war which could have affected the findings. It is important to mention that some registry‐based studies have evaluated the incidence of HF after acute coronary syndromes [15]. Future RCTs should investigate the role of SGLT2 inhibitors in this specific subset of patients as well.

The results of this meta‐analysis should be approached with caution due to some limitations. Firstly, this was a study‐level meta‐analysis without individual patient‐level data, hence individual differences in patient characteristics could not be explored. Secondly, the results of our meta‐analysis are driven by two large RCTs (DAPA‐MI and EMPACT‐MI), and data from additional RCTs is required to confirm our findings. Thirdly, all the trials included in this meta‐analysis had huge differences in the number of male versus female patients enrolled, with males having over 80% representation. Hence, the extrapolation of the results of this analysis to female sex could introduce bias in outcomes.

## 5 Conclusion

In conclusion, this meta‐analysis demonstrates the efficacy of SGLT2 inhibitors in reducing HHF in patients presenting with acute MI. Large‐scale, RCTs with better representation of females, along with longer follow‐ups are warranted to evaluate the robustness of SGLT2 inhibitors in this population.

## Author Contributions

**Mushood Ahmed:** conceptualization, methodology, software, writing – original draft, writing – review and editing. **Hritvik Jain:** writing – original draft, investigation, software, formal analysis, project administration. **Hira Javaid:** data curation, formal analysis, investigation, writing – original draft. **Areeba Ahsan:** methodology, validation, formal analysis, data curation. **Szabolcs Szilagyi:** writing – review and editing, visualization, validation. **Adeel Ahmad:** supervision, writing – review and editing. **Raheel Ahmed:** writing – review and editing, supervision, visualization.

## Ethics Statement

No ethical approval was required for the study.

## Conflicts of Interest

The authors declare no conflicts of interest.

## Supporting information


Appendix S1.

## Data Availability

All data generated or analysed during this study are included in this article. Further inquiries can be directed to the corresponding author.
