# Supplementary material for: Efficacy of Sodium‐Glucose Cotransporter‐2 Inhibitors in Patients With Acute Myocardial Infarction: A Meta‐Analysis of Randomised Controlled Trials
Source: Endocrinol Diabetes Metab. 2024 Aug 15;7(5):e514. doi: 10.1002/edm2.514 (PMC11325160; doi:10.1002/edm2.514)
Supplement: Supplementary file 1 — Appendix S1. [file EDM2-7-e514-s001.docx]

**Supplementary Appendix**

**Efficacy of Sodium‐Glucose Cotransporter‐2 Inhibitors in Patients With Acute Myocardial Infarction: A Meta-Analysis of Randomized Controlled Trials**

**Journal Name:** Endocrinology, Diabetes and Metabolism

Mushood Ahmed^1^, Hritvik Jain^2^, Hira Javaid^3^, Areeba Ahsan^4^, Szabolcs Szilagyi^5^, Adeel Ahmad^6^, Raheel Ahmed^7,8*^

^1^ Rawalpindi Medical University, Rawalpindi, Pakistan

^2^ All India Institute of Medical Sciences (AIIMS), Jodhpur, India

^3^ Allama Iqbal Medical College, Lahore, Pakistan

^4^ Foundation University School of Health Sciences, Islamabad, Pakistan

^5^ Department of Cardiology, Northumbria Hospitals NHS Foundation Trust, UK

^6^ Chelsea and Westminster Hospital, London, UK

^7^ Department of Cardiology, Royal Brompton Hospital, London, UK

^8^ National Heart & Lung Institute, Imperial College London, London, UK

***Corresponding author**

Raheel Ahmed

[R.ahmed21@imperial.ac.uk](mailto:R.ahmed21@imperial.ac.uk)

Royal Brompton Hospital

National Heart & Lung Institute, Imperial College London

Supplementary Figure 1: PRISMA flowchart showing the screening and study selection process


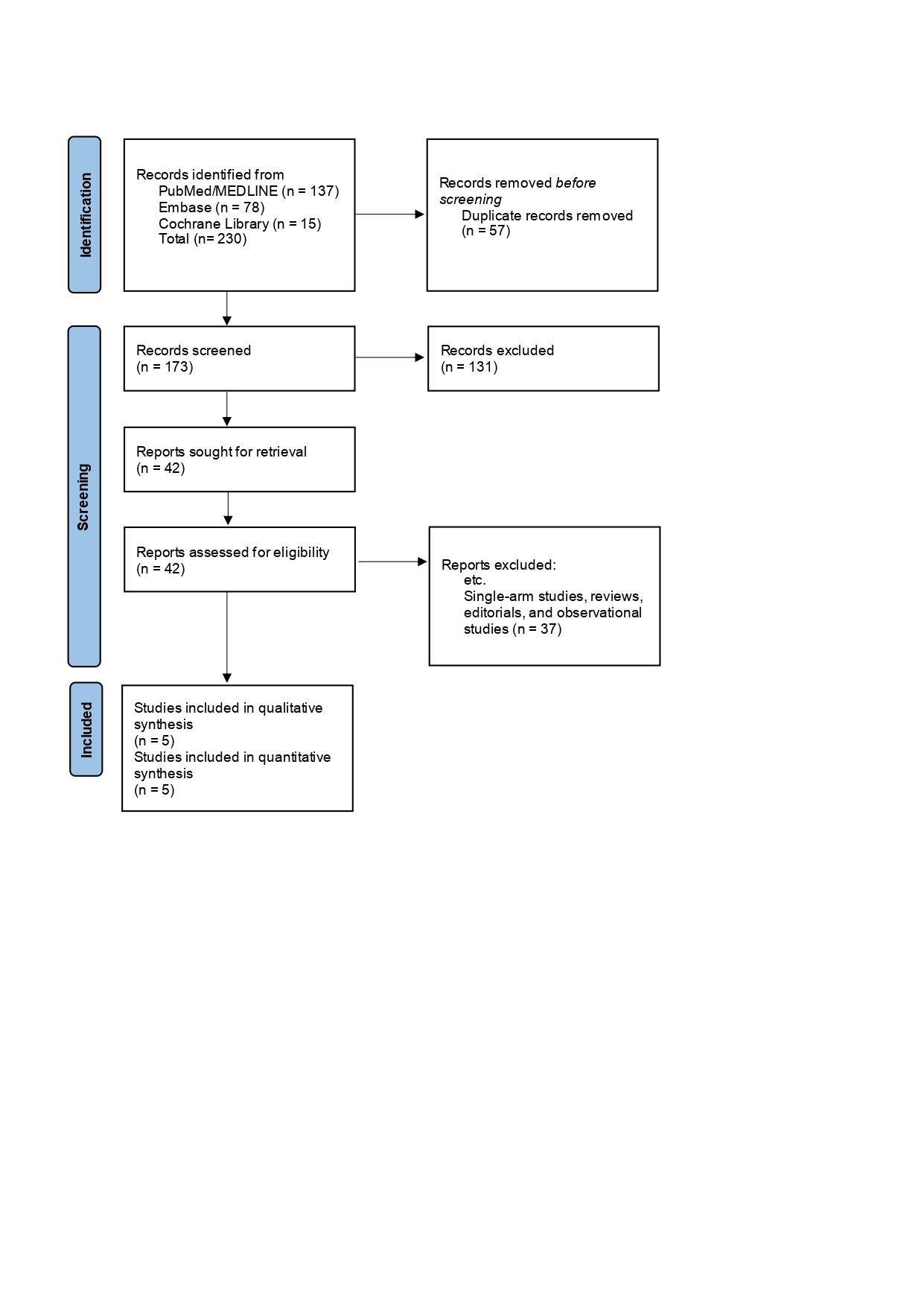


Supplementary Table 1: Inclusion and exclusion criteria in each study

| **Trial** | **Inclusion criteria** | **Exclusion criteria** |
| --- | --- | --- |
| **EMBODY**  **2020** | 1. Adults (aged ≥ 20 years), 2. Glycemic condition, Subjects appropriately diagnosed as T2DM by the Japanese guideline, Drug-naïve subjects or taking single anti-diabetic agent, T2DM patients who need to start or are possibly changing or adding an anti-diabetic agent. 3. Patients within 2–12 weeks after the onset of AMI, who can be discharged home | 1. Type 1 diabetes mellitus, 2. Persistent atrial fibrillation, 3.Insulin and glucagon-like peptide-1 analog user, 4. High dose of sulfonylurea (glimepiride > 2 mg, glibenclamide > 1.25 mg, glimicron > 40 mg), 5. HbA1c ≥ 10%, 6. History of diabetic ketoacidosis or diabetic coma within 3 months prior to the randomization, 7. Renal dysfunction (eGFR < 45 ml/min/1.73 m²), 8. Heart failure graded at NYHA functional class IV, 9. Pregnancy or possible pregnancy and breast feeding, 10. Lack of informed consent, 11. Contraindications to empagliflozin according to the label |
| **EMMY**  **2022** | Patients aged 18–80 years with a confirmed acute large MI (creatine kinase >800 IU/L), a high-sensitivity Troponin T level (or Troponin I level) >10-fold the upper limit of normal, and an estimated glomerular filtration rate >45 mL/min/1.73 m2 were eligible for inclusion. | Those with diabetes mellitus other than Type 2, a blood pH <7.32, hemodynamic instability, acute symptomatic urinary tract infection or genital infection, an ongoing SGLT2i treatment or an SGLT2i treatment within 4 weeks prior to enrolment, were excluded. |
| **DACAMI**  **2023** | Patients admitted with criteria for anterior ST-elevation myocardial infarction according to the fourth universal definition of myocardial infarction & show echocardiographic evidence of reduced LV ejection fraction <50% & have undergone successful reperfusion by primary percutaneous coronary angiography (pPCI). | (1) Patients with Diabetes Miletus Type 2 (DMT2), Type 1 (DMT1), secondary diabetes (e.g., endocrinopathies) (2) Patients already diagnosed with heart failure before this event (3) Patients on cardiotoxic chemotherapeutic medications. (4) Patients with hemoglobinopathies (5) Patients with chronic organ damage (i.e., chronic hepatitis with MELD score > 10, Stage 4 & 5 renal disease). (6) Patients already on SGLT2i. (7) Patients requiring additional anticoagulant therapy (i.e., patients with transthoracic echocardiographic evidence of left ventricular thrombus). (8) Patients with contraindications for the use of dapagliflozin, including patients with severely impaired renal function (eGFR <30 ml/min/1.73 m2) &/OR previous history of genitourinary infections (i.e.urosepsis, pyelonephritis & Fournier's gangrene) &/OR at high risk of such infections. |
| **DAPA MI**  **2023** | 1. Men or women age ≥18 at the time of signing the informed consent 2. Confirmed myocardial infarction (MI), either ST elevation MI or non-ST-elevation MI, according to the fourth universal definition of MI,2 within the preceding 7 days, or 10 days if earlier randomization is not feasible 3. Imaging evidence of impaired regional or global left ventricular (LV) systolic function at any timepoint during the index MI-related hospitalization (established with echocardiogram, radionuclide ventriculogram, contrast angiography or cardiac magnetic resonance imaging) OR definitive evidence on electrocardiogram of a Q-wave MI (defined as presence of Q waves in two or more contiguous leads, excluding leads III and aVR, and meeting all the following criteria: at least 1.5 mm in depth; at least 30 ms in duration; and, if R wave present, more than 25% of the size of the subsequent R wave) 4. Hemodynamically stable at randomization (no episodes of symptomatic hypotension, or arrhythmia with hemodynamic compromise in the last 24 hours). 5. Capable of giving signed informed consent that includes compliance with the requirements and restrictions listed in the informed consent form and in the protocol 6. Provision of signed and dated, written informed consent prior to any mandatory trial specific procedures, sampling, and analyses | 1. Known type 1 or type 2 diabetes at the time of admission. Patients with hyperglycemia, but without a diagnosis of diabetes mellitus prior to the index event, were eligible at the discretion of the investigator. 2. Chronic symptomatic heart failure (HF) with a prior hospitalization due to HF within the last year and known reduced LV ejection fraction (LVEF≤40 %), documented before the current MI hospitalization3. Severe chronic kidney disease (eGFR <20 mL/min/1.73 m2 by local laboratory), unstable or rapidly progressing kidney disease at the time of recruitment 4. Severe hepatic impairment (Child-Pugh class C) at the time of recruitment for the trial 5. Active malignancy requiring treatment at the time of screening, except for basal cell- or squamous cell carcinoma of the skin, presumed possible to treat successfully 6. Any non-CV condition, e.g. malignancy, with a life expectancy of less than two years based on the investigator ́s clinical judgement 7. Currently on treatment, or with an indication for treatment, with a sodium glucose co-transporter 2 inhibitor (SGLT2-inhibitor) 8. Known intolerance to dapagliflozin. 9. Participation in a) another trial with a non-approved investigational drug or blinded treatment with a CV or glucose lowering medication b) the planning and/or conduct of the trial (applies to AstraZeneca staff, Uppsala Clinical Research staff, and/or staff at the trial site) c) previous randomization in the present trial 10. Judgement by the investigator that the participant should not participate in the trial if the participant is unlikely to comply with trial procedures, restrictions and requirements, or any condition in the opinion of the Investigator that would make participation unsafe or unsuitable. 11. Women of childbearing potential (i.e., those who are not chemically or surgically sterilized or postmenopausal): a) Who are not willing to use a highly effective method of contraception, OR b) Who have a positive pregnancy test, OR c) Who are breast-feeding. |
| **EMPACT MI**  **2024** | Patients were men and women 18 years of age or older who had been hospitalized with an acute myocardial infarction within 14 days before randomization and had either evidence of a newly developed left ventricular ejection fraction of less than 45% or signs or symptoms of congestion that resulted in treatment during the index hospitalization (or both). Patients needed to have at least one additional enrichment factor (a clinical factor that was known to be associated with hospitalization for heart failure or death from any cause), including an age of 65 years or older; a newly developed ejection fraction of less than 35%; a history of myocardial infarction, atrial fibrillation, or type 2 diabetes; an estimated glomerular filtration rate (GFR) of less than 60 ml per minute per 1.73 m2 of body-surface area; an elevated natriuretic peptide or uric acid level; an elevated pulmonary artery or right ventricular systolic pressure; three-vessel coronary artery disease; peripheral artery disease; or no revascularization for the index myocardial infarction. | Patients with a previous diagnosis of heart failure, as well as those who were taking or planning to take SGLT2 inhibitors, were excluded. |

Supplementary Figure 2: Risk of bias graph and summary for the pooled trials


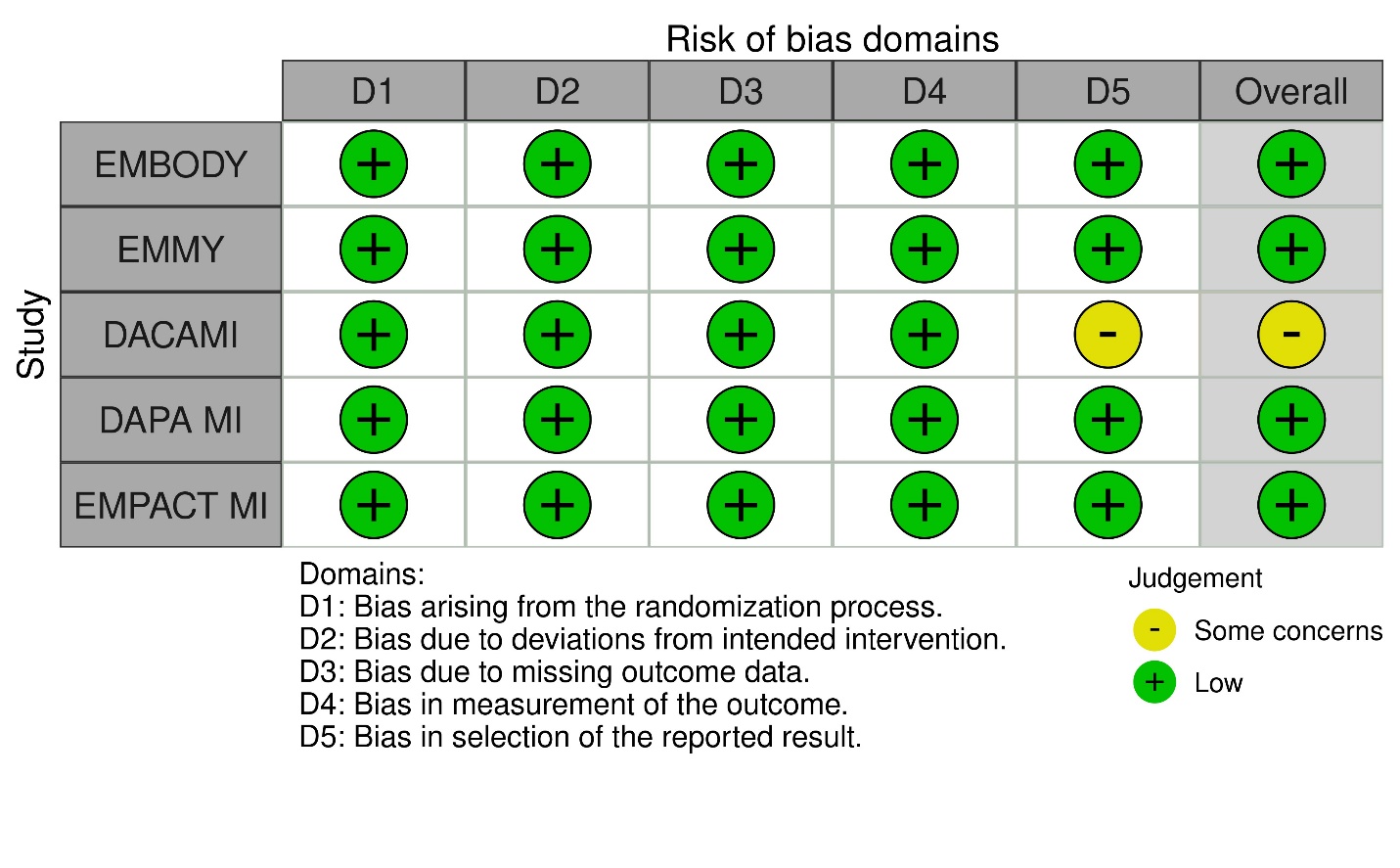


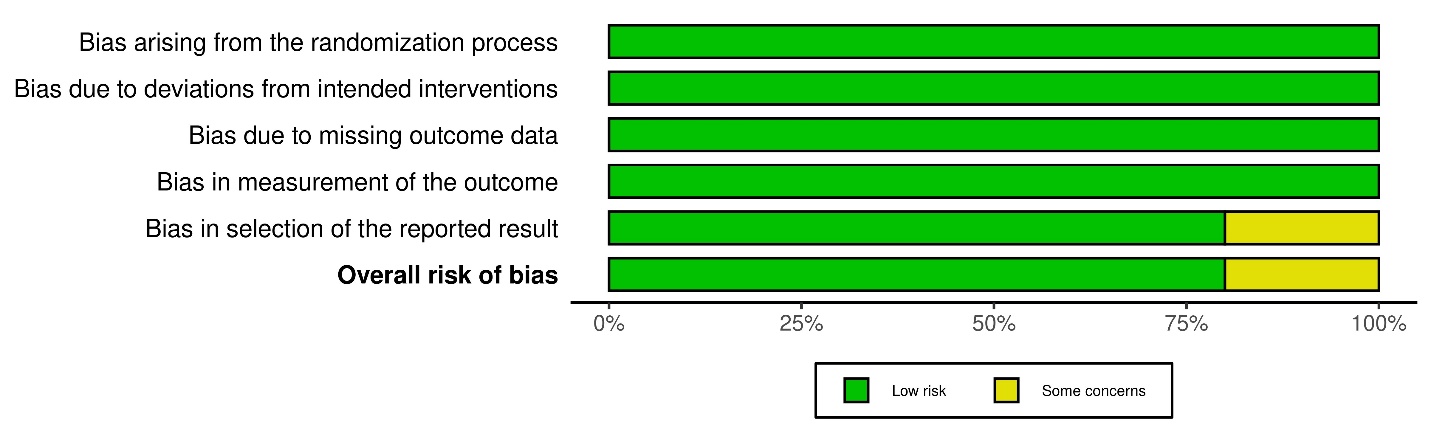


Supplementary Table 2: GRADE assessment for certainty of evidence and summary of findings table

|  | | | | | | | | | | | |
| --- | --- | --- | --- | --- | --- | --- | --- | --- | --- | --- | --- |
| **Certainty assessment** | | | | | | | **Summary of findings** | | | | |
| **Participants (studies) Follow-up** | **Risk of bias** | **Inconsistency** | **Indirectness** | **Imprecision** | **Publication bias** | **Overall certainty of evidence** | **Study event rates (%)** | | **Relative effect (95% CI)** | **Anticipated absolute effects** | |
|  |  |  |  |  |  |  | **With Placebo** | **With SGLT 2 Inhibitors** |  | **Risk with Placebo** | **Risk difference with SGLT 2 Inhibitors** |
| **All-Cause Mortality** | | | | | | | | | | | |
| 11211 (5 RCTs) | not serious | not serious | not serious | not serious | none | ⨁⨁⨁⨁ High | 211/5599 (3.8%) | 213/5612 (3.8%) | **RR 1.05** (0.78 to 1.41) | 38 per 1,000 | **2 more per 1,000** (from 8 fewer to 15 more) |
| **CV mortality** | | | | | | | | | | | |
| 11211 (5 RCTs) | not serious | not serious | not serious | not serious | none | ⨁⨁⨁⨁ High | 154/5599 (2.8%) | 161/5612 (2.9%) | **RR 1.04** (0.84 to 1.29) | 28 per 1,000 | **1 more per 1,000** (from 4 fewer to 8 more) |
| **HHF** | | | | | | | | | | | |
| 11211 (5 RCTs) | not serious | not serious | not serious | not serious | none | ⨁⨁⨁⨁ High | 247/5599 (4.4%) | 180/5612 (3.2%) | **RR 0.73** (0.61 to 0.88) | 44 per 1,000 | **12 fewer per 1,000** (from 17 fewer to 5 fewer) |
| **All-Cause Hospitalizations** | | | | | | | | | | | |
| 11211 (5 RCTs) | not serious | not serious | not serious | not serious | none | ⨁⨁⨁⨁ High | 619/5599 (11.1%) | 657/5612 (11.7%) | **RR 1.06** (0.96 to 1.17) | 111 per 1,000 | **7 more per 1,000** (from 4 fewer to 19 more) |

**CI:** confidence interval; CV; cardiovascular; HHF; hospitalizations for heart failure; **RR:** risk ratio;
